# Granulocyte-colony stimulating factor does not prevent in vitro cisplatin-induced germ cell reduction in immature human and mouse testis

**DOI:** 10.1186/s12885-023-10702-y

**Published:** 2023-03-16

**Authors:** Gabriele Matilionyte, Grace Forsyth, Jingtao Guo, Michael P. Rimmer, Brian P. Hermann, Richard A. Anderson, Rod T. Mitchell

**Affiliations:** 1 MRC Centre for Reproductive Health, The Queen’s Medical Research Institute, The University of Edinburgh, 47 Little France Crescent, Edinburgh, EH16 4TJ Scotland, UK; 2 State Key Laboratory of Stem Cell and Reproductive Biology, Institute of Zoology, Chinese Academy of Sciences, Beijing, 100101 China; 3 Beijing Institute for Stem Cell and Regenerative Medicine, Beijing, 100101 China; 4 University of Chinese Academy of Sciences, Beijing, 100049 China; 5 Department of Neuroscience, Developmental and Regenerative Biology, The University of Texas at San Antonio, 1 UTSA Circle, San Antonio, TX 78249 USA; 6 Royal Hospital for Children & Young People, 50 Little France Crescent, Edinburgh, EH16 4TJ Scotland, UK

**Keywords:** Human, Mouse, Testis, Cisplatin, Germ cell, Fertility preservation, Fetal, Pre-pubertal, G-CSF

## Abstract

**Background:**

Currently there are no established fertility preservation options for pre-pubertal boys facing cancer treatment. Granulocyte-colony stimulating factor (G-CSF) treatment has been proposed to be chemoprotective against spermatogonial cell loss in an alkylating chemotherapy model of busulfan treated adult mice. Having previously shown that exposure to the alkylating-like chemotherapy cisplatin resulted in a reduction in germ cell numbers in immature human testicular tissues, we here investigate whether G-CSF would prevent cisplatin-induced germ cell loss in immature human and mouse (fetal and pre-pubertal) testicular tissues.

**Methods:**

Organotypic in vitro culture systems were utilised to determine the effects of clinically-relevant concentrations of G-CSF in cisplatin-exposed immature testicular tissues. Human fetal (*n* = 14 fetuses) and mouse pre-pubertal (*n* = 4 litters) testicular tissue pieces were cultured and exposed to cisplatin or vehicle control for 24 hrs and analysed at 72 and 240 hrs post-exposure. Combined G-CSF and cisplatin exposure groups explored varying concentrations and duration of G-CSF supplementation to the culture medium (including groups receiving G-CSF before, during and after cisplatin exposure). In addition, effects of G-CSF supplementation alone were investigated. Survival of total germ cell and sub-populations were identified by expression of AP2γ and MAGE-A4 for human gonocytes and (pre)spermatogonia, respectively, and MVH and PLZF, for mouse germ cells and putative spermatogonial stem cells (SSCs) respectively, were quantified.

**Results:**

Exposure to cisplatin resulted in a reduced germ cell number in human fetal and mouse pre-pubertal testicular tissues at 240 hrs post-exposure. Germ cell number was not preserved by combined exposure with G-CSF using any of the exposure regimens (prior to, during or after cisplatin exposure). Continuous supplementation with G-CSF alone for 14 days did not change the germ cell composition in either human or mouse immature testicular tissues.

**Conclusions:**

This study demonstrates that exposure to G-CSF does not prevent cisplatin-induced germ cell loss in immature human and mouse testicular tissues in an in vitro system.

**Supplementary Information:**

The online version contains supplementary material available at 10.1186/s12885-023-10702-y.

## Background

Improvements in chemotherapeutic drug development have resulted in more than 80% of children with cancer surviving for more than 5 years [1]. Increasing survivorship means there is a growing population of childhood cancer survivors with unmet health needs, including treatment-induced infertility. It is well recognised that chemotherapeutic agents, notably alkylating agents, have off target effects throughout the body, including in the testis, which can lead to infertility in adult life [2–4]. Cisplatin is an alkylating-like platinum-based chemotherapeutic drug commonly used in paediatric oncology [5]. The understanding of how exposure to cisplatin affects the future fertility of pre-pubertal boys predominantly comes from retrospective follow-up studies [6]. The largest childhood cancer survivor cohort study to date demonstrated both males and females treated with cisplatin during childhood have a lower rate of pregnancy when they are adults compared to their siblings, who did not have childhood cancer [7]. Understanding how individual chemotherapeutic agents affect the immature male gonad comes from experimental studies using in vitro models. Cisplatin exposure to mouse pre-pubertal testicular tissues led to reduced germ cell number, particularly in the percentage of the proliferating germ cells [8–10]. Similar findings were demonstrated in immature human testicular tissues (fetal and pre-pubertal), which showed an acute reduction in total germ cell number in cisplatin-exposed tissues [11].

The key germ cell populations found in the human fetal testes are gonocytes (AP2γ^+ve^ cells) and (pre)spermatogonia, also known as (pro)spermatogonia or fetal spermatogonia (MAGE-A4^+ve^) [12]. Gonocytes and spermatogonia are present in the testis in infancy [12], whilst spermatogonia are the key germ cell population found in the pre-pubertal testis. Recent single cell sequencing of human fetal and pre-pubertal testis tissue demonstrated significant overlap in transcriptome of the germ cell populations [13, 14], making the human fetal testicular tissues a relevant surrogate model to explore the impact of exposure to pharmaceutical compounds during infancy and childhood.

The impact of chemotherapy treatment on immature testis is clear, however there are no established fertility preservation strategies for male pre-pubertal cancer patients. Current strategies are experimental and involve testicular tissue biopsy prior to chemotherapy treatment and then either maturing tissue or extracted cells in vitro or transplanting these back into the patient [15]. However, approaches to restore fertility using cryopreserved tissues have not been translated into human. In addition, these procedures require surgery that may not be possible should they delay life-saving treatment while re-transplanting tissues confers the potential risk of re-introducing malignant cells into the patient [1]. Therefore, there is an increasing need to develop chemoprotective agents to minimise chemotherapy-induced damage to the testis, without altering the efficacy of the cancer treatment itself. Based on animal studies, G-CSF has been identified as a potential candidate for protecting the testis from chemotherapy-induced damage. Adult male mice exposed to the chemotherapeutic agent busulfan that were co-treated with G-CSF were partially protected from loss of spermatogenesis [16]. A follow-up study demonstrated G-CSF treatment alone increased the spermatogenic capacity, suggesting spermatogenic regeneration in chemotherapy-induced loss of spermatogenesis [17]. In addition, adult female mice that were exposed to cisplatin and received G-CSF co-treatment had delayed onset of premature ovarian insufficiency compared to mice treated with cisplatin alone [18]. These studies suggest that G-CSF could protect adult mouse gonads from chemotherapy-induced damage. Recombinant human G-CSF (filgrastim) is used in clinical practice for mobilisation of haematopoietic progenitor cells in cancer patients with chemotherapy-induced neutropenia and those receiving chemotherapy prior to undergoing bone marrow transplantation [19]. This highlights the feasibility of G-CSF use in children with cancer should its efficacy in animal models be translated to humans.

There are no human studies investigating whether G-CSF could be used as a chemoprotective agent for pre-pubertal boys. Therefore, this study aimed to investigate whether exposure to one of several regimens of combined G-CSF and cisplatin could protect the key germ cell populations from cisplatin-induced damage in immature human testicular tissues, and to identify species-dependent effects of G-CSF exposure with and without cisplatin by comparing findings in human and mouse immature testicular tissues.

## Methods

### Experimental design

**Fig. 1:**
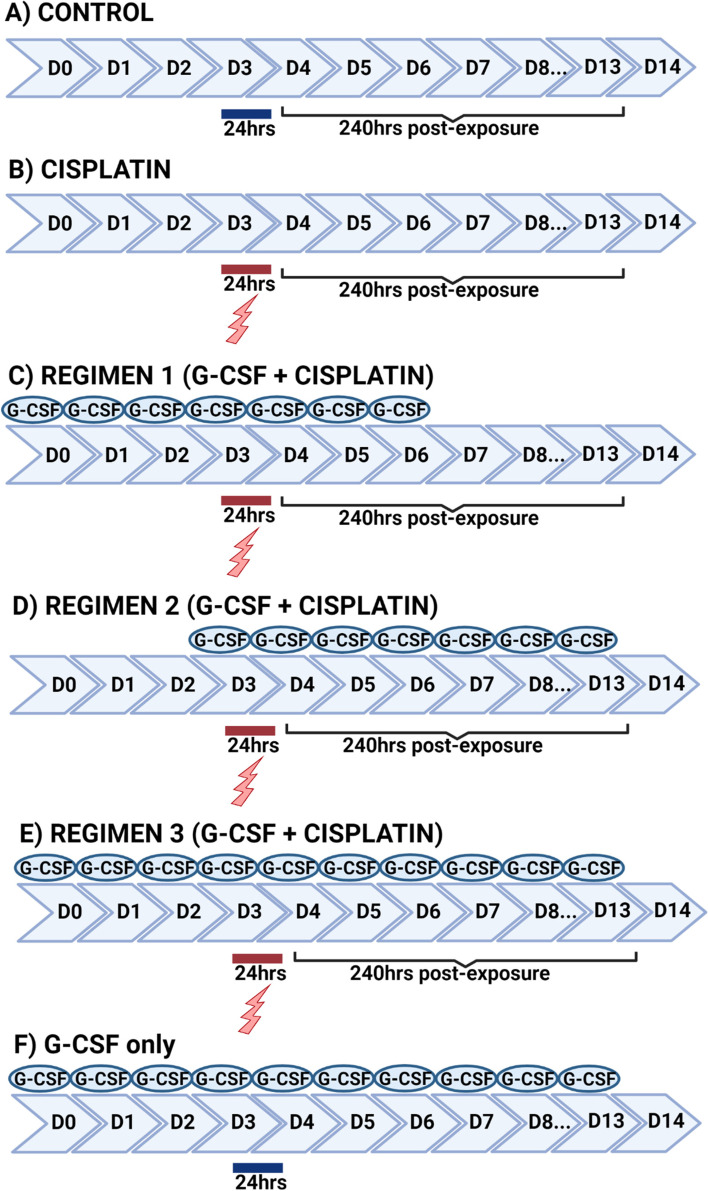
Schematic representation of treatments used in tissue culture experiments. Lightning bolt and red line represent when pieces were exposed to medium containing chemotherapy for 24 hrs. Blue line shows exposure to medium containing ddH2O for 24 hrs. Created with BioRender.com

Given that exposure to cisplatin results in germ cell loss in human fetal testicular tissues [11] and that treatment with G-CSF partially protected germ cells from chemotherapy (busulfan) induced damage in adult male mice [16, 17], we combined this knowledge and aimed to determine whether co-exposure to G-CSF would protect immature human germ cells from cisplatin-induced damage. We have previously demonstrated that exposure to clinically-relevant concentration(s) of cisplatin significantly reduced total germ cell number in human fetal testicular tissues with acute reduction seen in gonocyte population (at 72 hrs post-exposure) and delayed response in (pre)spermatogonial numbers (at 240 hrs post-exposure) [11]. We also demonstrated that effects on (pre)spermatogonia at 240 hours post-exposure were restricted to the late second trimester [20], hence we analysed testicular tissues from fetuses obtained at 17–22 Gestational Weeks (GW). Combined G-CSF and cisplatin regimens were adapted from studies using adult male mice [16, 17]. A schematic representation of all regimens is shown in Fig. 1.

In the initial set of experiments, human fetal testicular tissue pieces were cultured in a hanging drop system and exposed to a clinically-relevant concentration of cisplatin (0.5 μg/ml) or vehicle control on day 3 for 24 hrs. In combined G-CSF and cisplatin groups, tissue pieces were exposed to clinically-relevant doses of G-CSF (5 and 20 ng/ml) once daily for 7 days (from 3 days prior, during and until 3 days after cisplatin exposure) at the beginning of the culture. The tissue culture was ended at 240 hrs post-cisplatin exposure (14-day culture). This set of experiments is referred to as Regimen 1.

For the second set of experiments, Regimen 2, in combined G-CSF and cisplatin groups, human fetal testicular tissue pieces were cultured in regular culture medium for 3 days prior to cisplatin treatment and exposed to a range of G-CSF concentrations (5, 10, 20 and 100 ng/ml) daily starting on the day of cisplatin exposure until 10 days after chemotherapy exposure (240 hrs post-exposure, 14-day culture).

In the third set of experiments (Regimen 3), human fetal testicular tissue pieces were exposed to G-CSF throughout the whole 14-day culture period (from 3 days prior, during and until 240 hrs after cisplatin exposure).

In addition, where sufficient tissue was obtained, human testicular tissue pieces were exposed to G-CSF alone (5, 10, 20 and 100 ng/ml) daily for a period of 14-day culture.

In the final set of experiments, Regimen 3, mouse pre-pubertal testicular tissue pieces were cultured at a liquid-air interphase on a floating membrane. Tissue pieces were exposed to cisplatin alone or vehicle control and combined 100 ng/ml G-CSF and cisplatin in all three regimens as described above for human fetal testicular tissue experiments. Exposure to 100 ng/ml G-CSF alone was performed daily for a 14-day culture period.

In all experiments, each individual fetus or mouse litter were considered the experimental unit (n), whilst each tissue fragment taken from an individual fetus or litter was considered an experimental replicate. Two tissue pieces were included per treatment per fetus/litter. Two tissue sections per tissue piece (3 slides in from either end of the tissue block) were subjected to immunostaining and each immunostaining run included all treatment groups, positive and negative controls (pre-culture tissue with and without primary antibody, respectively) from the same fetus/litter. Sample sizes were based on the results of previous studies. Positively stained cells were manually counted per seminiferous area (mm^2^) with assessor blinded to treatment details.

### Study approval

For experiments using human fetal testicular tissues, women gave informed consent to donate tissue for research and this work was approved by respective ethics committees (Edinburgh: South East Scotland Research Ethics Committee (LREC08/S1101/1), Newcastle: NRES committee North East – Newcastle and North Tyneside 1 (08/H0906/21 + 5) and London: NRES Committee London – Fulham (18/10/0822).

For studies involving animals, specific approval, including ethical approval, was given by the UK Home Office. All procedures were carried out by the University of Edinburgh Bioresearch and Veterinary Services and performed in accordance with the Animal (Scientific procedures) Act 1986.

### Tissue collection

#### Human fetal testicular tissues

Human fetal testicular tissues from 2nd trimester (*n* = 14; ages: 17–22 GW) were obtained from elective termination of pregnancy. This was undertaken at the Royal Infirmary of Edinburgh and additional tissue samples were obtained from Human Developmental Biology Resource (HDBR) facilities in Newcastle and London. None of the samples were from termination of pregnancy due to fetal abnormalities. Gestational age was determined by ultrasound and direct measurement of foot length. A small piece of skin or limb was taken to confirm the male sex of each fetus by presence of sex-determining region Y (*SRY*) gene by qPCR.

#### Mouse pre-pubertal testicular tissues

Pre-pubertal mouse testicular tissues (*n* = 4 litters, 3 pups per litter) were collected from post-natal day 5 male pups (C57/B6 strain), which were bred within the University of Edinburgh animal facility. Male pups were euthanised by cervical dislocation. Testes were removed and placed in cold dissection medium consisting of Leibovitz L-15 supplemented with 3 mg/ml of bovine serum albumin (BSA; both Sigma Aldrich) and kept on ice during transportation.

### In vitro culture systems

#### Hanging drop culture

Hanging drop culture was set up as previously described [21]. This culture system has been shown to support the maintenance of human fetal testicular tissues for up to 14 days and its utility in determining the impact of a variety of exposures on germ cell survival has been validated [11, 20, 21]. Human fetal testicular tissues were cut into pieces (~ 1 mm^3^) and randomly placed into 30 μl droplets of pre-warmed media on the upturned lid of Petri dish. The lid was then inverted and placed back on the dish, which contained a small volume (10 ml) of Phosphate Buffered Saline (PBS; Corning) at the bottom to keep the humidity inside the dish. The dish was placed in an incubator set to 5% CO_2_ at 37 °C.

Regular culture medium composition for human fetal testicular tissues consisted of: Minimum Essential Medium α (MEMα; Lonza), 1x MEM non-essential amino acids (Thermo Fisher Scientific), 2 mM sodium pyruvate (Thermo Fisher Scientific), 2 mM L-glutamine (Life Technologies), 1x Insulin-Transferrin-Selenium (ITS; Sigma-Aldrich), 1x penicillin/streptavidin (Thermo Fisher Scientific) and supplemented with 10% fetal bovine serum (FBS; Life Technologies). Treatment medium was prepared by adding an appropriate volume of stock solution into regular medium. Tissue culture medium was replaced every 24 hrs.

#### Membrane culture

Membrane culture was set up as previously described [9]. Briefly, mouse pre-pubertal testes were cut into ~ 0.5 mm^3^ pieces and placed on a Whatman membrane (GE Healthcare) floating on pre-warmed 1 ml treatment culture medium consisting of MEM (Gibco), 10% Knockout Serum Replacement (KSR) and appropriate concentration of drug stock per well on a 24-well plate. Fragments were randomly allocated to treatment groups. The plate was incubated at 35 °C, 5% CO_2_. Tissue culture medium was changed every 24 hrs.

### Drug preparation

#### Cisplatin

Cisplatin concentration (0.5 μg/ml) was based on publications reporting plasma serum levels in paediatric cancer patients after administration of cisplatin [22]. Stock solution of 0.5 mg/ml was reconstituted in sterile water prior to dilution in regular culture medium to obtain the cisplatin treatment.

#### Granulocyte-colony stimulating factor (G-CSF)

Concentrations of G-CSF used were selected based on reported plasma levels in paediatric patients after G-CSF administration [23]. To prepare 10 μg/ml G-CSF stock, human recombinant G-CSF (PeproTech) was reconstituted in 0.1% BSA in sterile water. Stock was used to prepare working concentrations (5–100 ng/ml) by diluting in tissue culture medium.

### Tissue processing

At the conclusion of each experiment, tissue pieces were fixed in Bouin’s fluid (Clin-Tech) for 1 hr. and transferred to 70% ethanol. Post fixation, samples were paraffin-embedded, sectioned (5 μm) and assessed by H&E using standard protocol or used for immunohistochemistry as described below. Only samples that showed healthy tissue morphology (defined tubules, minimal apoptosis and germ cell presence) in pre-culture and vehicle controls were included in the analyses. Two sections from two replicates for each treatment were stained and analysed.

### Immunostaining

Double colourimetric immunohistochemistry and double immunofluorescence were performed as previously described [11]. Staining was performed for the expression of AP2γ and MAGE-A4 to detect gonocytes and (pre)spermatogonia, respectively, in human fetal testicular tissues. For mouse pre-pubertal testicular tissues, staining for MVH and PLZF was performed to detect germ cells and the SSC sub-population, respectively. Summary of key reagents and dilutions are provided in supplementary Table 1.

### Statistics

Statistical analysis was performed using GraphPad Prism 8 software (GraphPad Software Inc., USA). Data are presented as mean ± standard error of mean (SEM). For any of the tests used, statistical significance was considered where *p* < 0.05.

#### Human tissues

Two-way analysis of variance (ANOVA) was performed as treatment group and individual sample were considered as two individual variables to assess for overall significance. To compare for differences of means between treatment groups, multiple comparisons were tested using Bonferroni’s post hoc test.

#### Mouse tissues

Data sets were tested for normality using Shapiro-Wilk test, confirming a normal distribution of the data. As each litter of pups was genetically similar, differences of means between treatment groups were analysed using unpaired two tailed t-test (when two treatment groups were compared) or one-way ANOVA (when three or more treatment groups were compared). Bonferroni’s post hoc test was performed to assess for significance between treatment groups.

### Single-cell sequencing analysis

Datasets from single-cell RNA sequencing of human fetal and pre-pubertal testis [13, 14] were interrogated to define the cell populations present in the testis and to localise expression of *CSF3R*. Data are presented as t-SNE plots.

## Results

### Short-term supplementation with G-CSF in combined exposure did not prevent cisplatin-induced germ cell reduction in human fetal testicular tissues

**Fig. 2:**
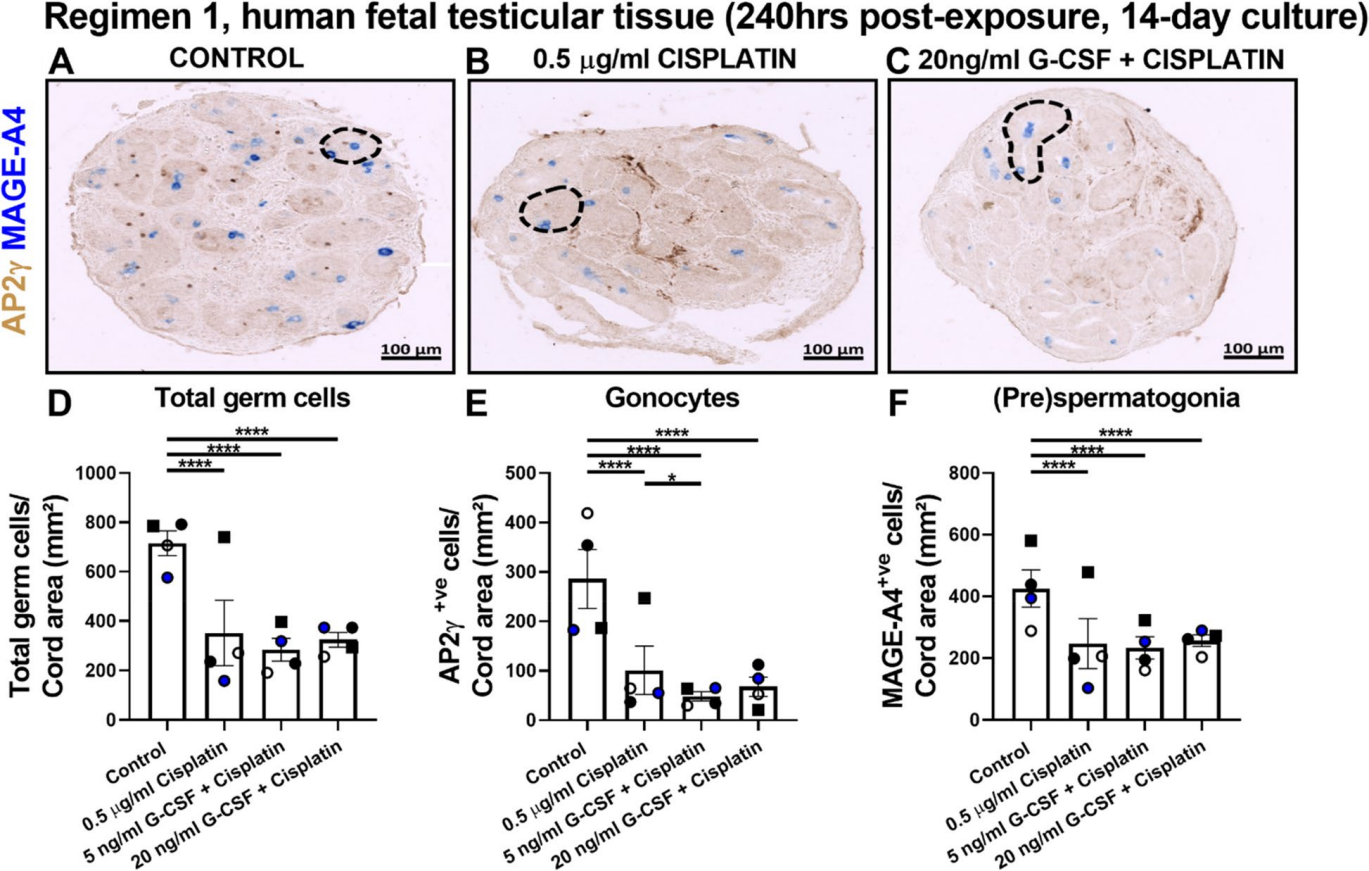
Effects of exposure to combined G-CSF and cisplatin (Regimen 1) compared to cisplatin alone or control on germ cell number in human fetal testicular tissues at 240 hrs post-exposure. Representative images of IHC staining for expression of gonocyte (AP2y, brown) and (pre)spermatogonial protein markers (MAGE-A4, blue) in control (**A**), 0.5 μg/ml cisplatin (**B**) or 20 ng/ml G-CSF + cisplatin (**C**) exposed human fetal testicular tissues at 240 hrs post-exposure. Dashed lines outline seminiferous cords. Quantification of total germ cell (**D**) and separate populations, gonocyte (**E**) and (pre)spermatogonial (**F**) numbers per cord area (mm^2^) at 240 hrs post-exposure. Each set of coloured points or squares represents an individual fetus (*n* = 3–4; 17-22GW). Data presented as mean ± SEM and analysed using two-way ANOVA (*p < 0.05, **p < 0.01, ****p < 0.0001)

In Regimen 1 (Fig. 2), human fetal testicular tissue pieces in combined G-CSF and cisplatin groups were exposed to 5 or 20 ng/ml G-CSF 3 days prior, during and 3 days after cisplatin exposure and germ cell analysis was performed at 240 hrs post-exposure (14-day culture). A significant reduction in total germ cell (352 ± 132 vs 715 ± 50 cells/cord area (mm^2^), *p* < 0.0001; Fig. 2D), gonocyte (101 ± 49 vs 286 ± 59, *p* < 0.0001, Fig. 2E) and (pre)spermatogonial cell (247 ± 81 vs 425 ± 60, *p* < 0.0001; Fig. 2F) numbers was found in cisplatin alone-exposed tissues compared to control. Reductions in total germ cell (Fig. 2D), gonocyte (Fig. 2E) and (pre)spermatogonial cell (Fig. 2F) numbers remained in tissues exposed to combined G-CSF and cisplatin compared to control. No difference in germ cell number was observed in combined G-CSF and cisplatin groups when compared to cisplatin alone except for exposure to 5 ng/ml G-CSF and cisplatin, where combined treatment resulted in significantly reduced gonocyte number compared to exposure to cisplatin alone (48 ± 9 vs 101 ± 49 cells/cord area (mm^2^), *p* < 0.05; Fig. 2E). These results indicated that short-term exposure (from 3 days prior, during and until 3 days after chemotherapy exposure) to 5 or 20 ng/ml G-CSF did not protect from cisplatin-induced germ cell loss in human fetal testicular tissues.

### Alternative regimens of G-CSF treatment did not prevent cisplatin-induced germ cell reduction in human fetal testicular tissues

**Fig. 3:**
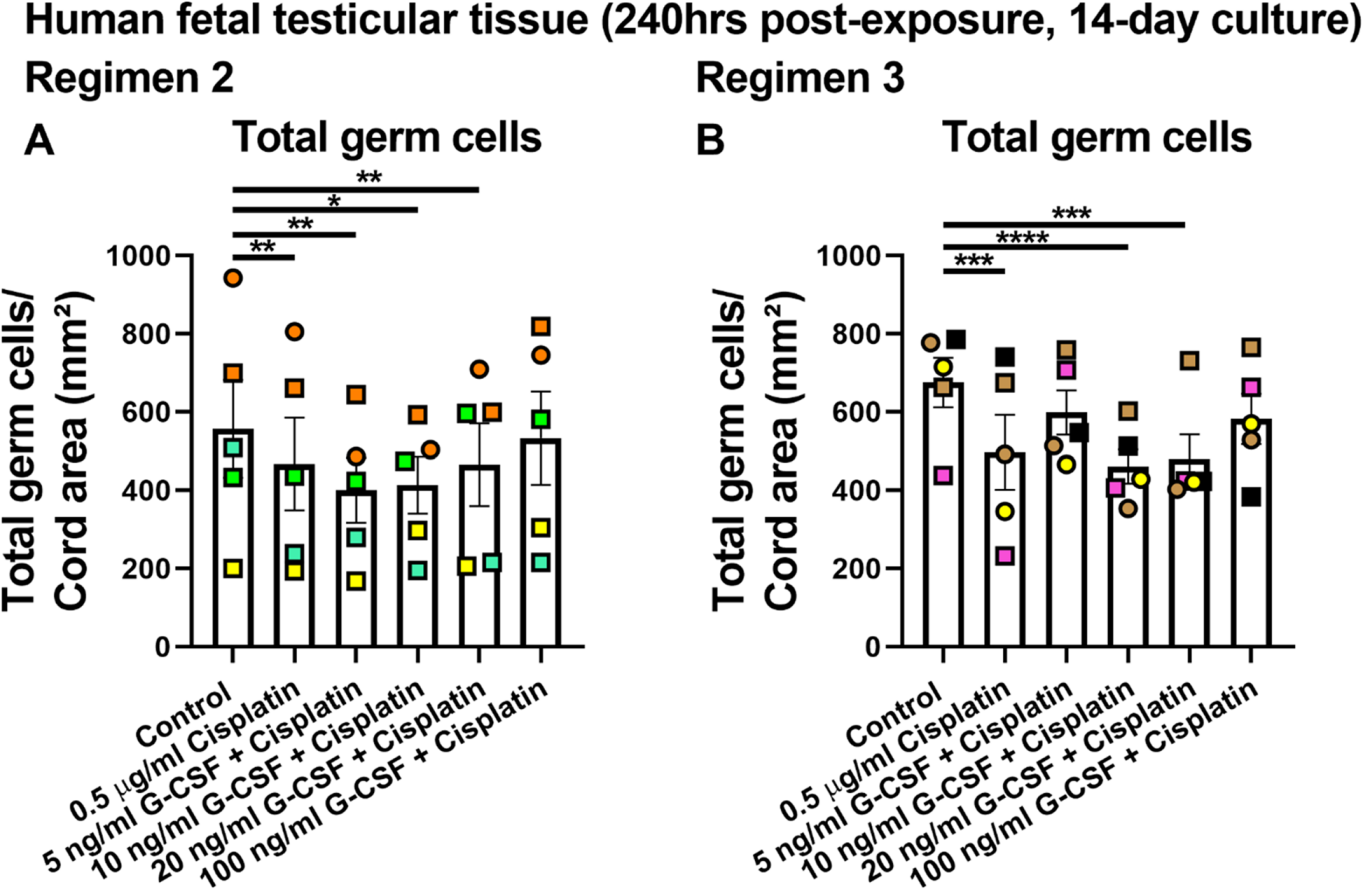
Effects of exposure to combined G-CSF and cisplatin (Regimen 2&3) compared to cisplatin alone or control on total germ cell number in human fetal testicular tissues at 240 hrs post-exposure. Quantification of total germ cell numbers per cord area (mm^2^) in tissues from Regimen 2 (**A**) and Regimen 3 (**B**). Each set of coloured points or squares represents an individual fetus (*n* = 4–5; 16-22GW). Data presented as mean ± SEM and analysed using two-way ANOVA (*p < 0.05, **p < 0.01, ***p < 0.001)

Given that short-term supplementation with G-CSF in combined treatment in Regimen 1 did not protect the human fetal testicular tissues from cisplatin-induced germ cell loss, alternative regimens and additional concentrations of G-CSF (5, 10, 20 and 100 ng/ml) in combined exposure experiments were investigated (Fig. 3). In Regimen 2, human fetal testicular tissue pieces were exposed to G-CSF during and for 10 days after cisplatin exposure (240 hrs post-exposure, 14-day culture). Exposure to cisplatin alone resulted in a significant reduction in total germ cell number (467 ± 118 vs 557 ± 125 cells/cord area (mm^2^), *p* < 0.01; Fig. 3A). Total germ cell number was also significantly reduced in 5, 10 and 20 ng/ml G-CSF and cisplatin groups compared to control (Fig. 3A). However, no difference in total germ cell number was seen between 100 ng/ml G-CSF and control. No change in total germ cell numbers was observed in any of the combined G-CSF and cisplatin exposure groups compared to cisplatin alone (Fig. 3A). In Regimen 3, human fetal testicular tissues were exposed to continuous G-CSF supplementation which included 3 days prior, during and 10 days after cisplatin exposure) (240 hrs post-exposure, 14-day culture) in the combined G-CSF and cisplatin groups. A significant reduction in total germ cell number (497 ± 96 vs 676 ± 64 cells/cord area (mm^2^), *p* < 0.001; Fig. 3B) was observed in cisplatin alone-exposed tissues compared to control. Total germ cell numbers were reduced in tissues exposed to 10 ng/ml G-CSF and cisplatin (461 ± 44 vs 676 ± 64, *p* < 0.0001) or 20 ng/ml G-CSF and cisplatin (480 ± 63 vs 676 ± 64, *p* < 0.001) but not in 5 or 100 ng/ml G-CSF and cisplatin exposure groups, compared to control (Fig. 3B). None of the combined treatments resulted in a significant difference in germ cell number compared to cisplatin alone. These results showed that exposure to alternative regimens of G-CSF in combined exposure did not protect from cisplatin-induced germ cell loss in human fetal testicular tissues.

In terms of separate germ cell sub-populations, exposure to cisplatin alone in Regimen 2 decreased the gonocyte number compared to control (145 ± 52 vs 222 ± 68 cells/cord area (mm^2^), *p* < 0.05; Fig. S1A). A significant reduction in gonocyte number was seen in all combined G-CSF and cisplatin exposure groups when compared to control but these numbers were not different when compared to cisplatin alone (Fig. S1A). No changes in (pre)spermatogonial numbers were seen between any of the exposure groups (Fig. S1B). In Regimen 3, a reduction in gonocyte number was observed in the 20 ng/ml G-CSF and cisplatin group compared to control (84 ± 13 vs 158 ± 11 cells/cord area (mm^2^), *p* < 0.01), but no differences were seen between any other exposure groups (Fig. S1C). The number of (pre)spermatogonia was significantly reduced in tissues exposed cisplatin alone (376 ± 85 vs 493 ± 57 cells/cord area (mm^2^), *p* < 0.05) or 10 ng/ml G-CSF and cisplatin compared to control (338.6 ± 30.6 vs 492.7 ± 57.2 cells/cord area (mm^2^), *p* < 0.01) but no changes were observed in any combined G-CSF and cisplatin groups compared to cisplatin alone (Fig. S1D).

### Exposure to G-CSF alone had no effect on the number of germ cells in human fetal testicular tissues

**Fig. 4:**
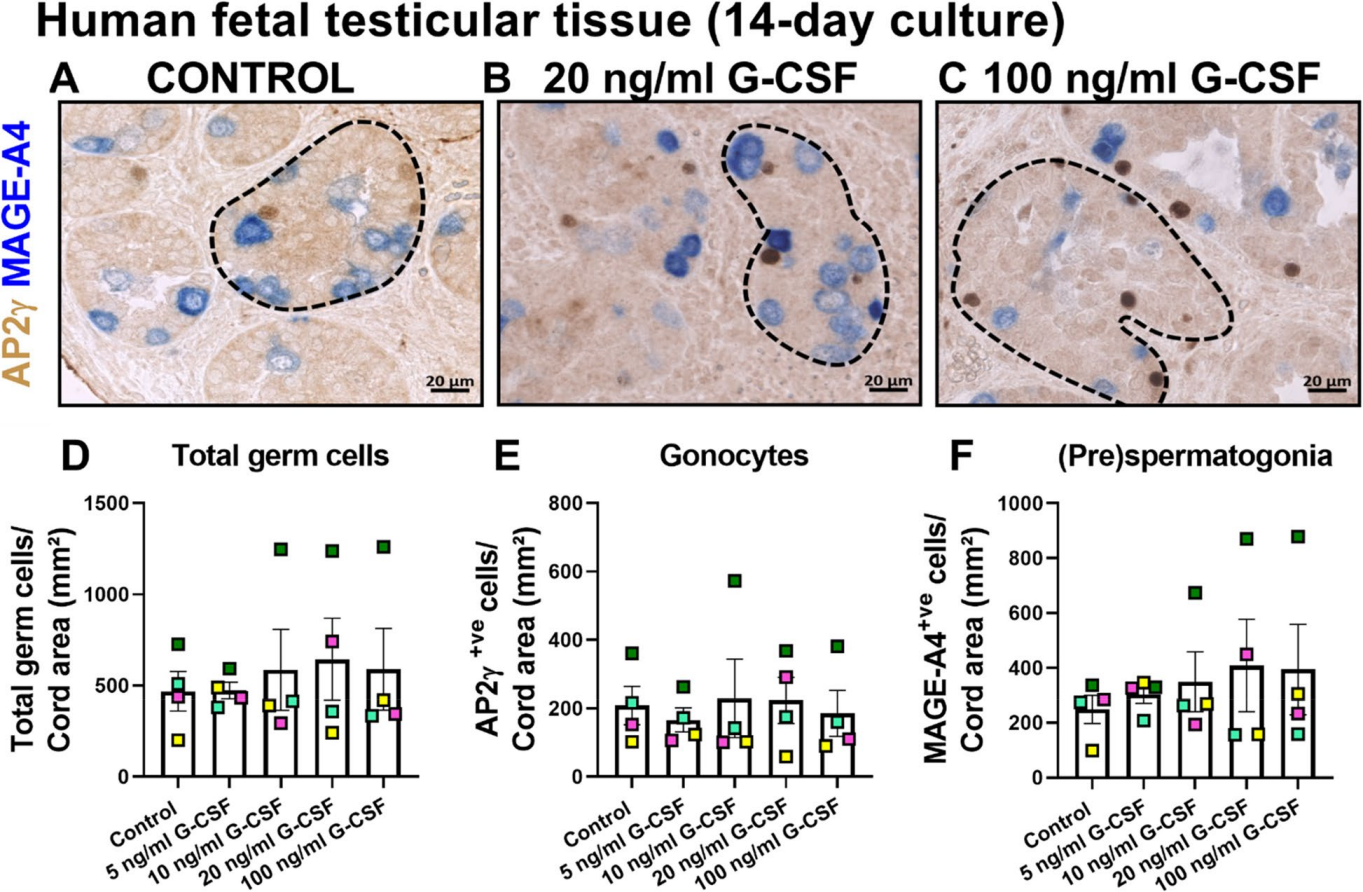
Effects of exposure to G-CSF alone for 14 days on germ cell number in human fetal testicular tissues. Representative images of IHC staining for expression of gonocyte (AP2y, brown) and (pre)spermatogonial protein markers (MAGE-A4, blue) in control (**A**), 20 ng/ml G-CSF (**B**) or 100 ng/ml G-CSF (**C**) exposed human fetal testicular tissues (17GW). Dashed lines outline seminiferous cords. Quantification of total germ cell (**D**) and separate populations, gonocyte (**E**) and (pre)spermatogonial (**F**) numbers per cord area (mm^2^) in tissues exposed to G-CSF for 14 days. Each colour represents an individual fetus (*n* = 4; 17-22GW). Data presented as mean ± SEM and analysed using two-way ANOVA (*p < 0.05)

A significant reduction in gonocyte number was observed in 5 ng/ml G-CSF and cisplatin compared to cisplatin-exposed group (Regimen 1, Fig. 2K) and total germ cell numbers were not different between 100 ng/ml G-CSF and cisplatin, compared to control-exposed group (Regimens 2&3, Fig. 3). We sought to understand whether exposure to G-CSF, without presence of cytotoxic insult, had an effect on germ cell number in human fetal testicular tissues. Sections from human fetal testicular tissue pieces exposed to 5, 10, 20 or 100 ng/ml G-CSF alone for 14 days (Fig. 4) were stained for the expression of AP2γ and MAGE-A4 to detect gonocytes and (pre)spermatogonia, respectively. Quantification of positively stained cells per cord area (mm^2^) showed daily exposure to a range of G-CSF concentrations (5–100 ng/ml) for 14 days had no effect on total germ cell, gonocyte or (pre)spermatogonial cell numbers (Fig. 4D-F).

### Exposure to G-CSF did not protect the germ cells from cisplatin-induced damage in mouse pre-pubertal testicular tissues

Results from experiments using immature human testicular tissues suggested that G-CSF did not show chemoprotective capabilities in contrast to published studies in adult male mice. To address the possibility of species-dependent action of G-CSF, culture experiments similar to those described for human tissues were performed using mouse pre-pubertal testicular tissues.

**Fig. 5:**
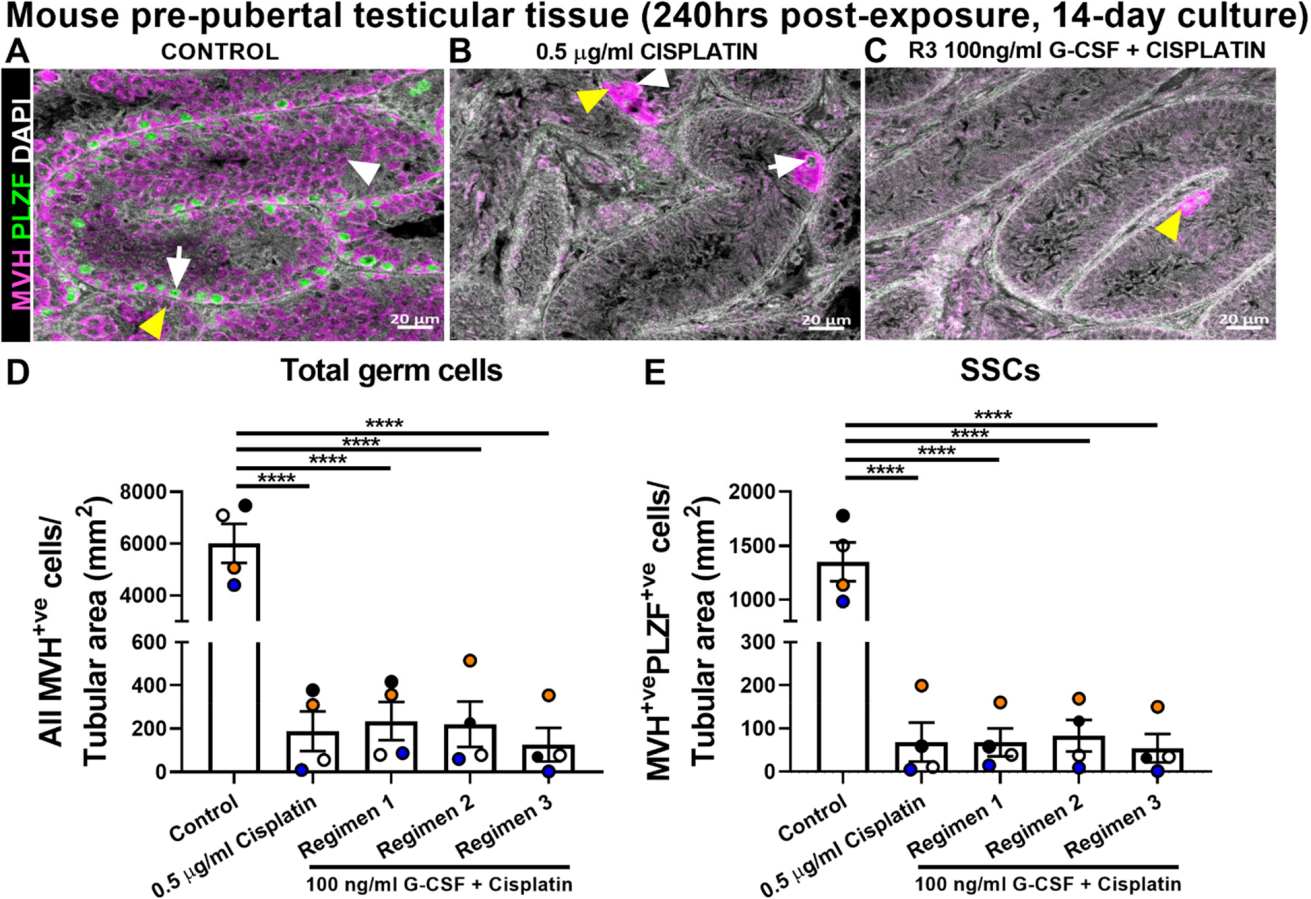
Effects of exposure to combined G-CSF + cisplatin on germ cell numbers in mouse pre-pubertal testicular tissues at 240 hrs post-exposure. Representative images of IF staining for expression of spermatogonial (MVH, purple) and SSC (PLZF, green) protein markers in control (**A**), 0.5 μg/ml cisplatin (**B**) or 100 ng/ml G-CSF + cisplatin exposed mouse pre-pubertal testicular tissues from Regimen 3 (**C**). White arrows point to MVH^+ve^PLZF^+ve^ cells, yellow arrowheads MVH^+ve^PLZF^-ve^ cells at the basement membrane and white arrowheads to MVH^+ve^PLZF^-ve^ cells in the middle of the tubules. Quantification of total germ cell (**D**) and putative SSC (**E**) numbers per tubular area (mm^2^). Each set of coloured points represents an individual litter (*n* = 4). Data presented as mean ± SEM and analysed using one-way ANOVA (****p < 0.0001)

Sections from tissue pieces exposed to control, cisplatin alone or combined 100 ng/ml G-CSF and cisplatin at 240 hrs post-exposure (14-day culture) using 3 different regimens were subjected to germ cell analysis based on the expression of MVH (all germ cells) and PLZF (putative SSCs) (Fig. 5A-C). At 240 hrs post-exposure (14-day culture), cisplatin significantly reduced the total germ cell number compared to tissues exposed to control (188 ± 91 vs 6009 ± 752 cells/tubular area (mm^2^), *p* < 0.0001; Fig. 5D). No difference in total germ cell number was observed between cisplatin alone and combined G-CSF and cisplatin exposure groups in all three regimens (Fig. 5D). Similarly, tissues exposed to cisplatin alone contained a significantly lower number of putative SSCs compared to control (69 ± 45 vs 1350 ± 180 cells/tubular area (mm^2^), *p* < 0.0001; Fig. 5E). Tissues exposed to combined G-CSF and cisplatin in all three regimens had a significantly reduced number of SSCs present compared to control (Fig. 5E). No difference in SSCs numbers were observed in combined G-CSF and cisplatin-exposed tissues compared to cisplatin alone exposure group (Fig. 5E). These results suggest that exposure to G-CSF as part of combined G-CSF and cisplatin treatment did not protect germ cells from cisplatin-induced damage in mouse pre-pubertal testicular tissues.

Previous animal studies suggested that exposure to G-CSF alone without chemotherapy exposure increased steady-state spermatogenesis in adult mice [17]. Tissue pieces of mouse pre-pubertal testes were exposed to 100 ng/ml G-CSF alone for 14 days to understand if a similar effect would be observed in pre-pubertal testicular tissues. No difference in total germ cell (Fig. S2C) or putative SSC (Fig. S2D) numbers were observed in tissues exposed to G-CSF alone compared to control, suggesting that exposure to G-CSF alone does not affect germ cell number in mouse pre-pubertal testicular tissues.

## Discussion

The main aim of this study was to understand whether exposure to G-CSF had a protective effect against cisplatin-induced reduction of germ cell numbers in immature testicular tissues, in both rodent and human. The results confirmed that cisplatin alone resulted in a significant reduction in total germ cell number but showed that this was not prevented by various regimens of G-CSF supplementation in combination, e.g. short-term supplementation with G-CSF or continuous exposure to G-CSF throughout the culture period. In addition, short supplementation with 5 ng/ml G-CSF in combined treatment in Regimen 1 resulted in a reduction in gonocyte number compared to cisplatin alone which could suggest that gonocyte sensitivity to cisplatin exposure may even be enhanced by G-CSF supplementation. However, it should also be pointed out that the cisplatin-only treatment group contained an outlier that may have influenced this result, which would also explain why a similar result was not seen with 20 ng/ml G-CSF. The Food and Drug Administration (FDA) datasheet for Filgrastim (G-CSF) states that exposure to G-CSF is thought to increase the sensitivity of immune cells to chemotherapy treatment in patients, thus G-CSF is not recommended to be given prior to chemotherapy treatment and that G-CSF administration should commence 24 hrs post-exposure to chemotherapy [24]. Should future studies find G-CSF to have potential to protect from chemotherapy-induced damage, administration would need reflect this aspect of clinical practice.

Exposure to cisplatin alone did not always result in a significant reduction in numbers of separate germ cell sub-populations, thus it cannot be clearly concluded whether supplementation with G-CSF in Regimens 2 and 3 were able to protect against cisplatin-induced damage in separate germ cell sub-populations. This could be explained by changes in germ cell composition during the development of human fetal testis. With increasing gestational age, the number of gonocytes decreases whilst the (pre)spermatogonial number increases [12, 25]. In addition, we utilised human fetal testicular tissues as a surrogate for investigating effects on human pre-pubertal testicular tissues. The key germ cell populations found in the human fetal testes are gonocytes (AP2γ^+ve^ cells) and (pre)spermatogonia, also known as (pro)spermatogonia or fetal spermatogonia (MAGE-A4^+ve^) [12]. Gonocytes and spermatogonia are present in the testis in infancy [12], whilst spermatogonia are the key germ cell population found in the pre-pubertal testis. Recent single cell sequencing of human fetal and pre-pubertal testis tissue demonstrated significant overlap in transcriptome of the germ cell populations making the human fetal testicular tissues a relevant surrogate model to explore the impact of exposure to pharmaceutical compounds during infancy and childhood [13, 14]. Therefore, future experiments may be performed using human pre-pubertal testicular tissues where spermatogonia are the key germ cell population. Whilst the activity of the hypothalamo-pituitary-gonadal axis of differs between the fetal (active axis) and prepubertal (quiescent axis) periods, the cultures are conducted without supplementation with gonadotrophins in order to simulate the situation in prepuberty.

Whilst this study was not intended to try and replicate the previous animal studies that reported chemoprotective effects of G-CSF in adult male mice exposed to busulfan [16, 17], it is interesting to note that we did not find any protection of germ cells from chemothaerpy-induced damage. These findings may be explained by fundamental and purposeful differences between the current study design and previously published work. This includes differences in chemotherapy agent, species, developmental stage and experimental approach. In the mouse study, adult mice (5 weeks of age) were injected with a single intraperitoneal dose of busulfan at 44 mg/kg of body weight and tissue analysed at 8 days, 10 or 19 weeks post-exposure [16, 17]. In this study, human fetal testicular tissues were exposed to 0.5 μg/ml cisplatin in vitro and analysis performed at 72 and 240 hrs post-exposure (7-day and 14-day culture, respectively). Busulfan is an alkylating drug whilst cisplatin is classified as alkylating-like; however, both drugs act by forming DNA adducts, thus, causing cell death [26]. Effects of exposure to busulfan on murine testicular tissues are well described including a study showing that injection with 30 mg/kg of body weight of busulfan resulted in azoospermia at 56 days post-treatment [27]. This study also showed that it was specifically the SSCs that were affected, with a lower impact observed on the remainder of the spermatogonia [27]. In terms of human exposure, busulfan is classified as resulting in one of the highest risks of infertility in childhood cancer survivors [4, 28]. Exposure to cisplatin during childhood has been shown to result in a lower chance of pregnancy in both females and males compared to their healthy siblings [26]. Our recent study showed that it could be due to direct targeting of SSCs in human pre-pubertal testicular tissues, where a significant reduction in putative SSCs (MAGE-A4^+ve^UTF1^+ve^ cells) was observed in tissues exposed to cisplatin compared to controls [11]. Taken together, it appears that both busulfan and cisplatin have similar impact on the germ cells in immature testicular tissues, therefore, the contrasting results with other G-CSF studies might not have been due to different chemotherapeutic agents used. In future studies, the potential of G-CSF as a chemoprotective agent against cisplatin-induced damage could be investigated alongside exposure to busulfan in order to confirm whether any chemotherapy specific effects are seen.

An alternative explanation for the differing results relates to species difference between findings reported in the animal studies and in this study. A recent publication investigated the effects of G-CSF on the reproductive function of male pre-pubertal guinea pigs and ram lambs [29]. Animals received daily injection of G-CSF for 4 days and assessment was performed 2-months following the treatment. G-CSF treatment resulted in an increase in testosterone levels in ram lambs but remained unchanged in guinea pigs [29], demonstrating species differences in testicular response to G-CSF. To address differences in species and developmental stage between the current study and published work we performed similar experiments using pre-pubertal mouse testicular tissues. Cultured tissues were exposed to control, 0.5 μg/ml cisplatin-only or combined G-CSF and cisplatin using the same regimens as for human fetal testicular tissue experiments. Results showed that at 240 hrs post-exposure (14-day culture), total germ cells and SSCs were significantly reduced in cisplatin alone-exposed tissues. These data are in agreement with the findings from several studies using murine pre-pubertal testicular tissues which showed that in vitro exposure to clinically-relevant concentrations of cisplatin led to similar degree of reduction in total germ cell and SSC numbers as seen in this study [8–10]. Of note, 8 weeks post-exposure the quantification of total germ cell or SSCs numbers showed no difference between cisplatin- and control- exposed tissues, suggesting a partial recovery of germ cells over a prolonged period in culture in the mouse [9], whereas germ cell loss following in vitro exposure to cisplatin in human fetal testis was maintained over a 12 week xenografting period [11].

A significant reduction in total germ cell and SSC number was seen in combined G-CSF and cisplatin exposed mouse pre-pubertal testicular tissues compared to control whereas no difference was observed when comparing to exposure to cisplatin alone at 240 hrs post-exposure (14-day culture), indicating that G-CSF exposure did not protect the testis from cisplatin-induced germ cell loss. In in vivo mouse experiments, the extent of the protective effects of G-CSF co-exposure with busulfan was minimal [16, 17] and the recovery of fertility in adult mouse testes was dependent on the threshold of remaining SSCs present after busulfan treatment; functional recovery of fertility occurred when number of SSCs equated to roughly 30% of the number that was present pre-treatment (day 0) [30]. The present study was designed to identify direct effects of G-CSF on the immature human testis and whether this could protect from cisplatin-induced germ cell loss. Therefore, the presence of the receptor for G-CSF, namely CSF3R, within the testis is particularly important. The study by Benavides-Garcia and colleagues, which showed partial protection from busulfan-induced damage, proposed a direct effect of G-CSF treatment on spermatogonial cells [16]. This was supported by data showing expression of CSF3R on isolated THY1^+ve^ spermatogonia. However, single-cell sequencing of human fetal and post-natal testis has shown the expression of *CSF3R* in testicular macrophages and not in the SSC population (Fig. S3). Taken together, our results indicate that G-CSF does not act directly on germ cells and does not prevent their loss in the immature mouse or human testis from cisplatin-induced damage. Given the findings of in vivo studies in adult mice, it remains possible that protection of germ cells could occur directly and indirectly as a result of a local effect on macrophages in the testis or systemic response to G-CSF, respectively. The latter point is supported by a study reporting increased production of GnRH and testosterone in ram lambs and guinea pigs exposed to G-CSF [29]. Further studies involving in vivo exposure of pre-pubertal mice to chemotherapy with and without G-CSF could be used to test this hypothesis. Furthermore, xenografting immature human testis tissues and exposing host mice to treatment can also be used to better reflect the in vivo effects of exposure, compared to in vitro approaches [31].

## Conclusions

This study has demonstrated in vitro exposure to G-CSF did not confer direct protection of immature mouse or human testicular tissues from cisplatin-induced germ cell loss. Future work to determine the role of G-CSF in protecting germ cells in the pre-pubertal testis from chemotherapy-induced damage should focus on other experimental models (e.g. in vivo, xenograft) capable of identifying indirect mechanisms that may result in protection of germ cells in the testis.

## Supplementary Information

**Additional file 1: Fig. S1.** Effects of exposure to combined G-CSF and cisplatin (Regimen 2&3) compared to cisplatin alone or control on gonocyte and (pre)spermatogonial numbers in human fetal testicular tissues at 240 hrs post-exposure. **Fig. S2.** Effects of exposure to G-CSF alone for 14 days on germ cell numbers in mouse pre-pubertal testicular tissue. **Fig. S3.** Expression of CSF3R in human fetal and post-natal testicular tissues.

**Additional file 2: Table S1.** Summary of immunohistochemistry (IHC) and immunofluorescent (IF) protocols.

## Data Availability

The data sets used and/or analysed during the current study are available from the corresponding author on reasonable request.

## Abbreviations

ANOVA: Analysis of variance
BSA: Bovine serum albumin
FBS: Fetal bovine serum
FDA: Food and Drug Administration
G-CSF: Granulocyte-colony stimulating factor
GW: Gestational weeks
HDBR: Human Developmental Biology Resource
ITS: Insulin-transferrin-selenium
KSR: Knockout serum replacement
MAGE-A4: Melanoma-associated antigen 4
MEM: Minimal essential medium
PBS: Phosphate buffered saline
PLZF: Promyelocytic leukemia zinc finger
SEM: Standard error of mean
SRY: Sex determining region on Y chromosome
SSC: Spermatogononial stem cells
UTF1: Undifferentiated Embryonic Cell Transcription Factor 1
