# Supplementary material for: Granulocyte-colony stimulating factor does not prevent in vitro cisplatin-induced germ cell reduction in immature human and mouse testis
Source: BMC Cancer. 2023 Mar 16;23:251. doi: 10.1186/s12885-023-10702-y (PMC10018904; doi:10.1186/s12885-023-10702-y)
Supplement: Supplementary file 1 — Additional file 1: Fig. S1. Effects of exposure to combined G-CSF and cisplatin (Regimen 2&3) compared to cisplatin alone or control on gonocyte and (pre)spermatogonial numbers in human fetal testicular tissues at 240 hrs post-exposure. Fig. S2. Effects of exposure to G-CSF alone for 14 days on germ cell numbers in mouse pre-pubertal testicular tissue. Fig. S3. Expression of CSF3R in human fetal and post-natal testicular tissues. [file 12885_2023_10702_MOESM1_ESM.docx]

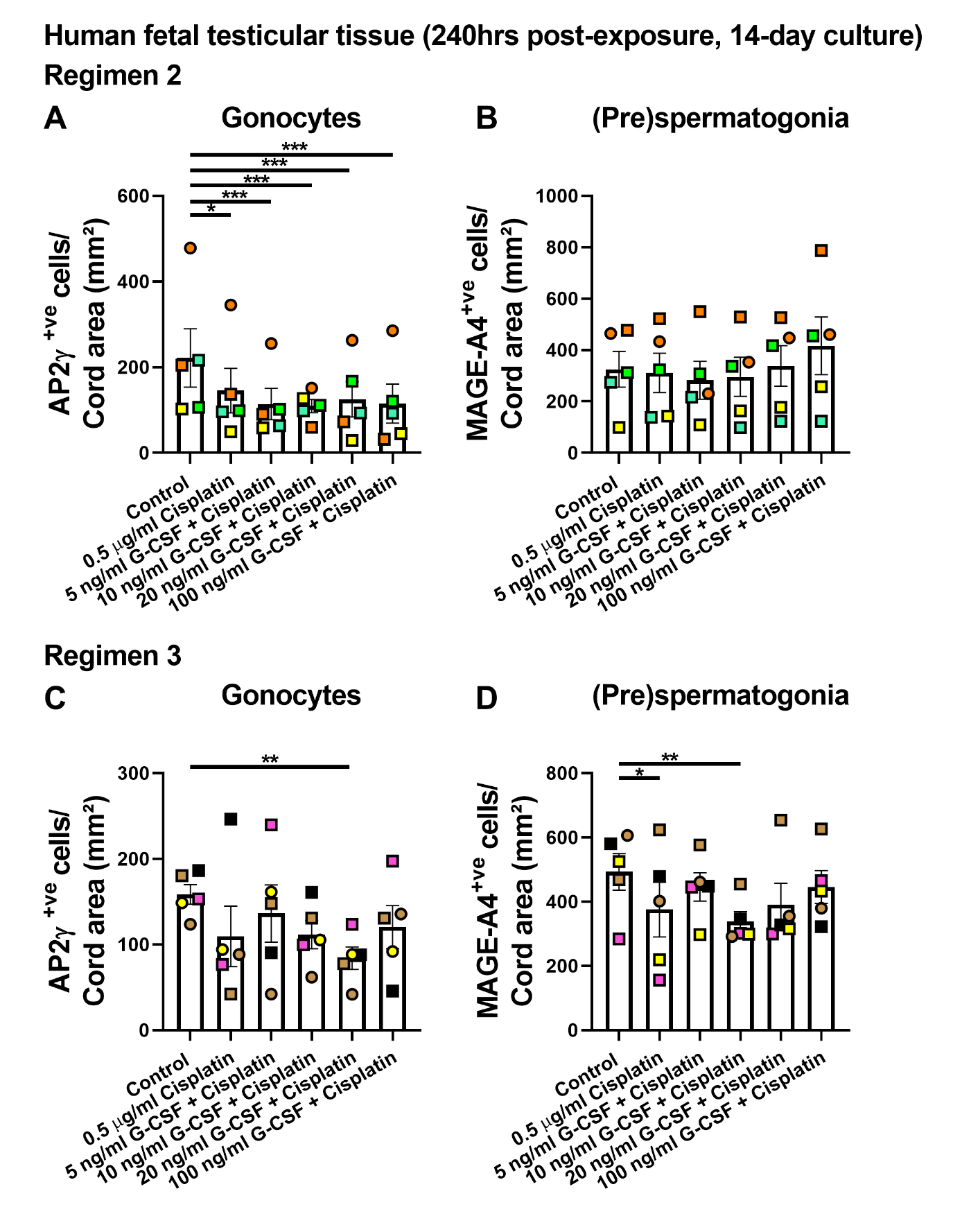


**Figure S1. Effects of exposure to combined G-CSF + cisplatin (Regimen 2&3) compared to cisplatin alone or control on gonocyte and (pre)spermatogonial numbers in human fetal testicular tissues at 240hrs post-exposure.** Quantification of gonocyte (A,C) and (pre)spermatogonial (B,D) numbers per cord area (mm^2^). Each set of coloured points or squares represents an individual fetus (n=5; 16-22GW). Data presented as mean±SEM and analysed using two-way ANOVA (*p<0.05, **p<0.01, ***p<0.001).


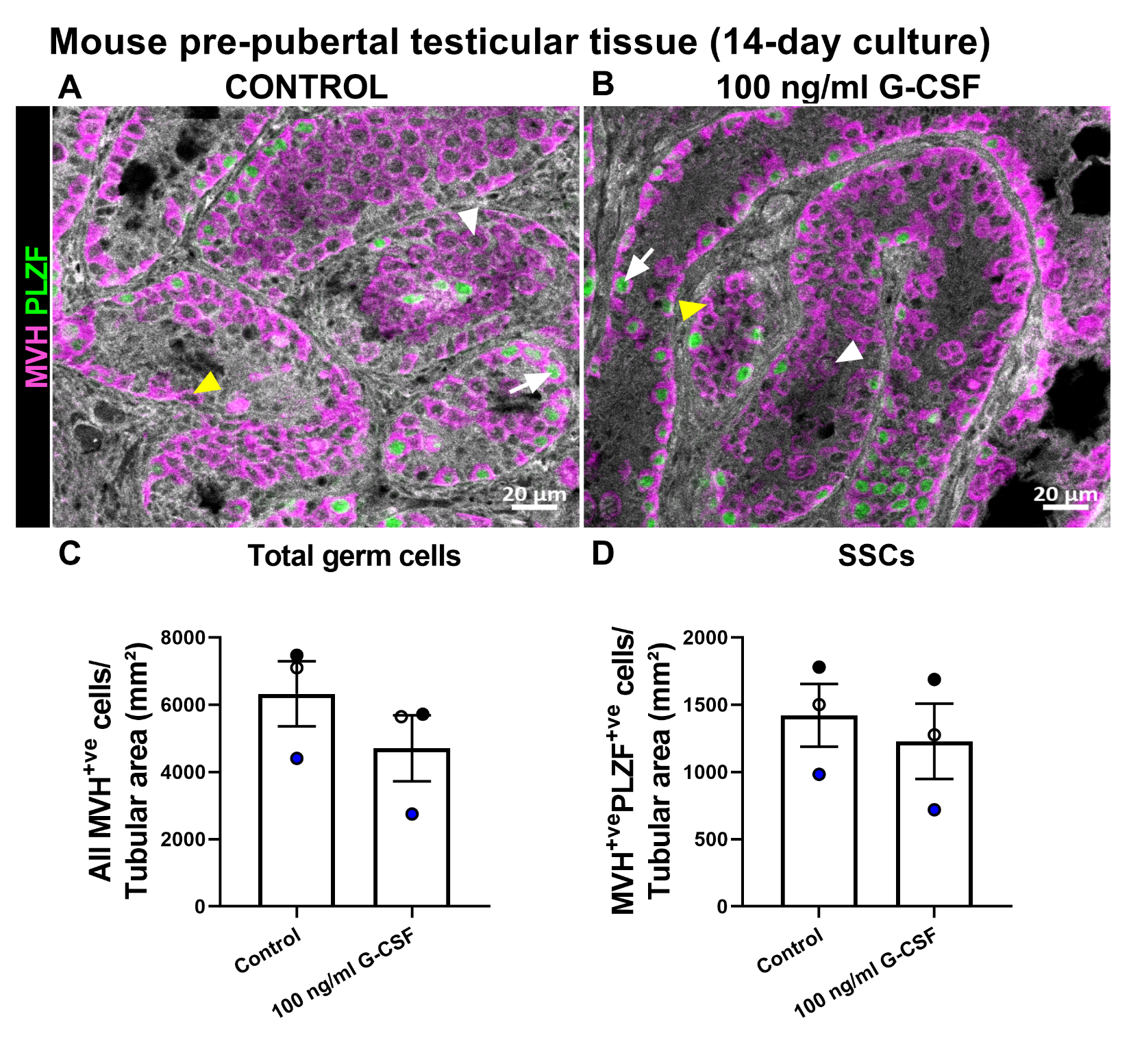


**Figure S2. Effects of exposure to G-CSF alone for 14days on germ cell numbers in mouse pre-pubertal testicular tissue.** Representative images of IF staining for spermatogonial (MVH, purple) and SSC (PLZF, green) protein markers in control (A) or 100 ng/ml G-CSF (B) exposed mouse pre-pubertal testicular tissues. White arrows point to MVH^+ve^PLZF^+ve^ cells, yellow arrowheads MVH^+ve^PLZF^-ve^ cells at the basement membrane and white arrowheads to MVH^+ve^PLZF^-ve^ cells in the middle of the tubules. Quantification of total germ cell (C) and putative SSC (D) numbers per tubular area (mm^2^). Each set of coloured points represents tissue pieces obtained from an individual litter (n=3). Data presented as mean±SEM and analysed using unpaired t-test.


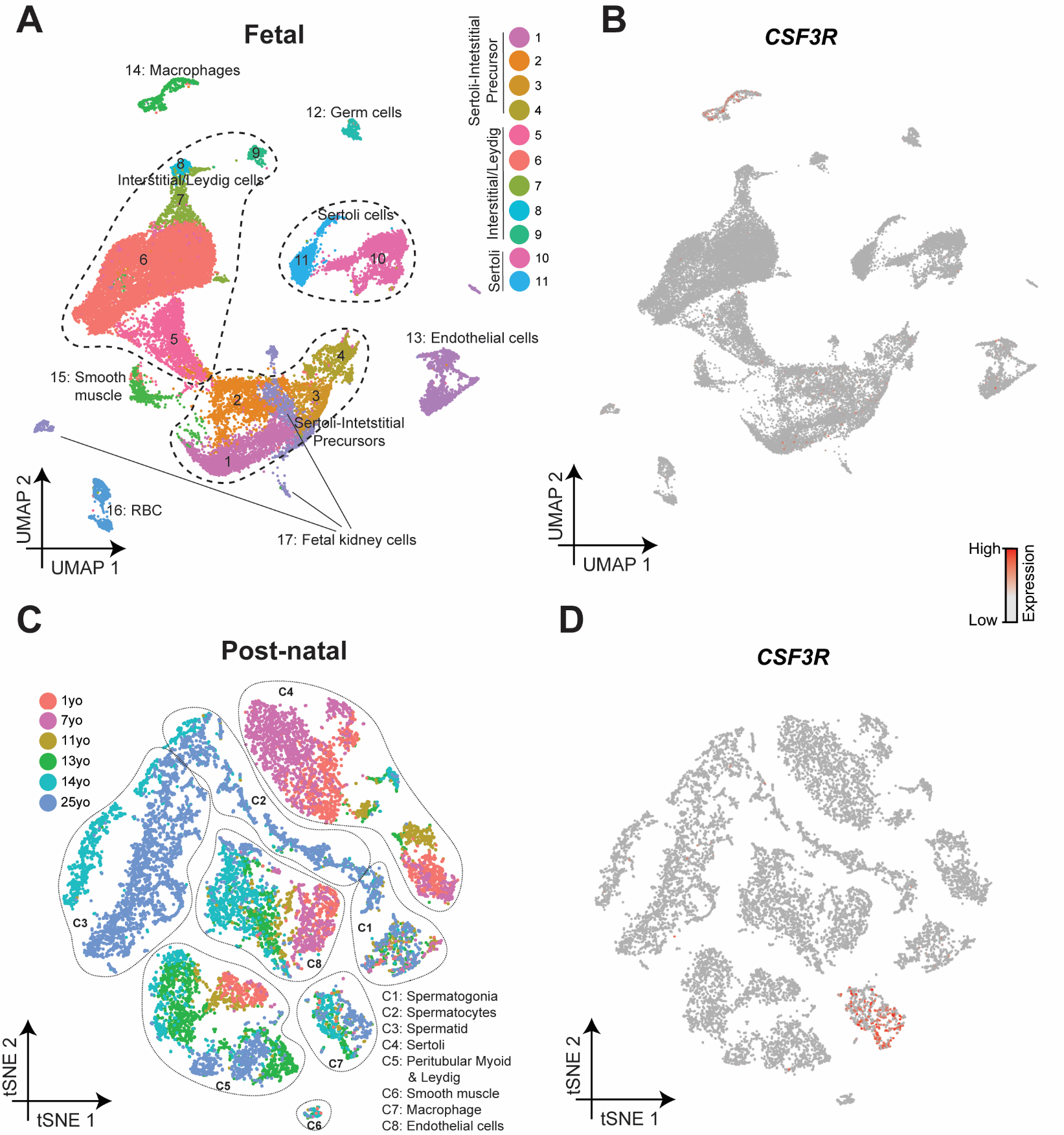


**FIGURE S3. Expression of *CSF3R* in human fetal and post-natal testicular tissues.** Clusters of cell populations in human fetal (A) and post-natal (C) testicular tissues and maps of *CSF3R* expression (B and D, respectively).
