# Supplementary material for: Granulocyte-colony stimulating factor does not prevent in vitro cisplatin-induced germ cell reduction in immature human and mouse testis
Source: BMC Cancer. 2023 Mar 16;23:251. doi: 10.1186/s12885-023-10702-y (PMC10018904; doi:10.1186/s12885-023-10702-y)
Supplement: Supplementary file 2 — Additional file 2: Table S1. Summary of immunohistochemistry (IHC) and immunofluorescent (IF) protocols. [file 12885_2023_10702_MOESM2_ESM.docx]

| **Antibody**  **(Cat no)** | **Method** | **Dilution**  **(antigen retrieval)** | **Origin** | **Blocking agent** | **Detection** |
| --- | --- | --- | --- | --- | --- |
| **AP2γ**  (sc-12762) | IHC | 1:20  (citrate buffer) | Mouse | Normal horse serum/  TBS/BSA | DAB |
| **MAGE-A4** (Gift from Giulio Spagnoli) | IHC | 1:40  (citrate buffer) | Mouse | Normal horse serum/  TBS/BSA | Vector Blue |
| **PLZF**  (Sc-28319) | IF | 1:50  (Tris-EDTA) | Mouse | Normal goat serum/  TBS/BSA | Tyramide-FITC |
| **MVH**  (Ab13840) | IF | 1:400  (citrate buffer) | Rabbit | Normal goat serum/  TBS/BSA | Tyramide-Cy3 |

**Supplementary Table 1**. Summary of immunohistochemistry (IHC) and immunofluorescent (IF) protocols. TBS – Tris-buffered Saline; BSA – Bovine Serum Albumin; DAB – 3,3’ di-amino-benzidene; FITC - Fluorescein isothiocyanate.
